# 2-Amino­pyridinium 4-hydroxy­benzoate

**DOI:** 10.1107/S1600536808034934

**Published:** 2008-10-31

**Authors:** Ching Kheng Quah, Samuel Robinson Jebas, Hoong-Kun Fun

**Affiliations:** a X-ray Crystallography Unit, School of Physics, Universiti Sains Malaysia, 11800 USM, Penang, Malaysia

## Abstract

In the title compound, C_5_H_7_N_2_^+^·C_7_H_5_O_3_^−^, the carboxyl­ate mean plane of the 4-hydroxy­benzoate anion is twisted by 8.78 (5)° from the attached ring. The cations and anions are linked *via* O—H⋯O, N—H⋯O and C—H⋯O hydrogen bonds, forming a three-dimensional network. In addition, π–π inter­actions involving the benzene and pyridinium rings, with centroid–centroid distances of 3.5500 (6) and 3.6594 (6) Å, are observed.

## Related literature

For the applications of 2-amino­pyridine, see: Windholz (1976 ▶). For related structures, see: Chao *et al.* (1975 ▶); Heath *et al.* (1992 ▶); Jebas & Balasubramanian (2006 ▶); Joanna & Zaworotko (2005 ▶); Smith *et al.* (2000 ▶).
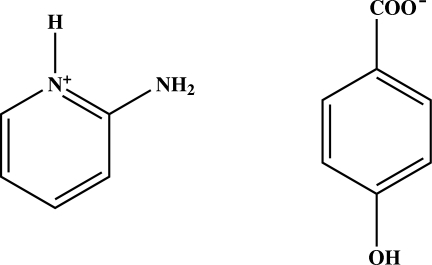


## Experimental

### 

#### Crystal data

C_5_H_7_N_2_^+^·C_7_H_5_O_3_^−^
*M* *_r_* = 232.24
Monoclinic, 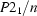

*a* = 10.0647 (2) Å
*b* = 10.9369 (2) Å
*c* = 10.7985 (2) Å
β = 111.036 (1)°
*V* = 1109.44 (4) Å^3^
*Z* = 4
Mo *K*α radiation
μ = 0.10 mm^−1^
*T* = 100.0 (1) K
0.34 × 0.29 × 0.17 mm

#### Data collection

Bruker SMART APEXII CCD area-detector diffractometer
Absorption correction: multi-scan (*SADABS*; Bruker, 2005 ▶) *T*_min_ = 0.966, *T*_max_ = 0.983
22398 measured reflections
5078 independent reflections
3835 reflections with *I* > 2σ(*I*)
*R*_int_ = 0.029

#### Refinement

*R*[*F* ^2^ > 2σ(*F* ^2^)] = 0.049
*wR*(*F* ^2^) = 0.140
*S* = 1.02
5078 reflections
158 parameters
1 restraint
H atoms treated by a mixture of independent and constrained refinement
Δρ_max_ = 0.50 e Å^−3^
Δρ_min_ = −0.21 e Å^−3^

### 

Data collection: *APEX2* (Bruker, 2005 ▶); cell refinement: *APEX2* and *SAINT* (Bruker, 2005 ▶); data reduction: *SAINT*; program(s) used to solve structure: *SHELXTL* (Sheldrick, 2008 ▶); program(s) used to refine structure: *SHELXTL*; molecular graphics: *SHELXTL*; software used to prepare material for publication: *SHELXTL* and *PLATON* (Spek, 2003 ▶).

## Supplementary Material

Crystal structure: contains datablocks global, I. DOI: 10.1107/S1600536808034934/ci2696sup1.cif

Structure factors: contains datablocks I. DOI: 10.1107/S1600536808034934/ci2696Isup2.hkl

Additional supplementary materials:  crystallographic information; 3D view; checkCIF report

## Figures and Tables

**Table 1:** Hydrogen-bond geometry (Å, °)

*D*—H⋯*A*	*D*—H	H⋯*A*	*D*⋯*A*	*D*—H⋯*A*
O1—H1*O*1⋯O2^i^	0.82	1.86	2.6257 (9)	154
N2—H1*N*2⋯O2^ii^	0.86	1.98	2.8224 (11)	167
N2—H2*N*2⋯O3^iii^	0.86	1.99	2.8396 (11)	171
N1—H1*N*1⋯O3^ii^	0.88 (1)	1.81 (1)	2.6861 (10)	169 (2)
C10—H10*A*⋯O1	0.93	2.51	3.3482 (14)	149
C11—H11*A*⋯O2^i^	0.93	2.34	3.1899 (12)	152

Symmetry codes: (i) 
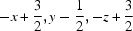
; (ii) 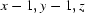; (iii) 
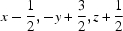
.

**Fig. 1.**
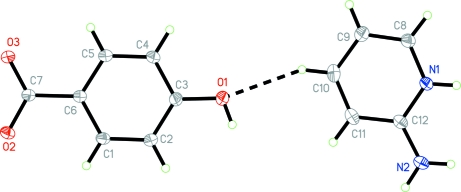
The molecular structure of the title compound, showing 50% probability displacement ellipsoids and the atom numbering scheme. The dashed line indicates a hydrogen bond.

**Fig. 2.**
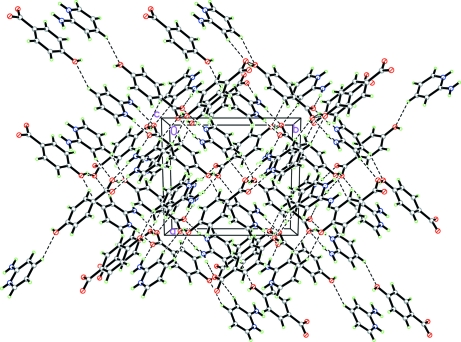
The crystal packing of the title compound, viewed along the c axis. Hydrogen bonds are shown as dashed lines.

## supplementary crystallographic information

### Comment

2-Aminopyridine is used in the manufacture of pharmaceuticals, especially antihistaminic drugs (Windholz, 1976). As an extension of our systematic study of hydrogen bonding patterns of 2-aminopyridine with aromatic carboxylic acids, the title compound was synthesized and its crystal structure determined.

The asymmetric unit contains one 2-aminopyridinium cation and 4-hydroxybenzoate anion. The proton transfer from the carboxyl group to atom N1 of 2-aminopyridine resulted in the widening of C8—N1—C12 angle of the pyridinium ring to 122.35°, compared to the corresponding angle of 117.7 (1)° in neutral 2-aminopyridine (Chao *et al.*, 1975). Similar feature is observed in various 2-aminopyridine acid complexes (Joanna & Zaworotko, 2005; Smith *et al.*, 2000). The bond distances and angles in the title compound are comparable to those in various 2-aminopyridine acid complexes and 4-hydroxybenzoic acid (Joanna & Zaworotko, 2005; Heath *et al.*, 1992).

The 2-aminopyridinium cation is essentially planar, with a maximum deviation of 0.016 (1) Å for atom N1. In the 4-hydroxybenzoate anion, the carboxylate group is twisted slightly from the attached ring; the dihedral angle between C1-C6 and O2/O3/C6/C7 planes is 8.78 (5)°.

The crystal packing is consolidated by intermolecular O—H···O, N—H···O and C—H···O hydrogen bonds (Table 1). These hydrogen bonds link the molecules into a three-dimensional network. The packing is further strengthened by π-π interactions involving the benzene (centroid Cg1) and pyridinium (centroid Cg2) rings, with Cg1···Cg2^iv^ = 3.6594 (6) Å and Cg2···Cg2^v^ = 3.5500 (6) Å [symmetry code: (iv) 1-x, 1-y, 1-z; (v) -x, 1-y, 1-z].

### Experimental

2-Aminopyridine and 4-hydroxybenzoic acid were mixed in methanol in a 1:1 molar ratio. The clear colourless solution obtained was allowed to evaporate slowly. Colourless crystals were obtained after a week.

### Refinement

H atoms were placed in calculated positions, with C-H = 0.93 Å, N-H = 0.86 Å and O-H = 0.82 Å and refined using a riding model with *U*_iso_(H) = 1.2U_eq_(C,N) and 1.5U_eq_(O). Atom H1N1 was located in a difference map and was refined with an N-H distance restraint of 0.86 Å.

### Crystal data

**Table:**

C_5_H_7_N_2_^+^·C_7_H_5_O_3_^−^	*F*(000) = 488
*M**_r_* = 232.24	*D*_x_ = 1.390 Mg m^−^^3^
Monoclinic, *P*2_1_/*n*	Mo *K*α radiation, λ = 0.71073 Å
Hall symbol: -P 2yn	Cell parameters from 6837 reflections
*a* = 10.0647 (2) Å	θ = 2.8–35.6°
*b* = 10.9369 (2) Å	µ = 0.10 mm^−^^1^
*c* = 10.7985 (2) Å	*T* = 100 K
β = 111.036 (1)°	Block, colourless
*V* = 1109.44 (4) Å^3^	0.34 × 0.29 × 0.17 mm
*Z* = 4	

### Data collection

**Table:**

Bruker SMART APEXII CCD area-detector diffractometer	5078 independent reflections
Radiation source: fine-focus sealed tube	3835 reflections with *I* > 2σ(*I*)
graphite	*R*_int_ = 0.030
φ and ω scans	θ_max_ = 35.6°, θ_min_ = 2.4°
Absorption correction: multi-scan (SADABS; Bruker, 2005)	*h* = −16→11
*T*_min_ = 0.966, *T*_max_ = 0.983	*k* = −17→17
22398 measured reflections	*l* = −16→17

### Refinement

**Table:**

Refinement on *F*^2^	Primary atom site location: structure-invariant direct methods
Least-squares matrix: full	Secondary atom site location: difference Fourier map
*R*[*F*^2^ > 2σ(*F*^2^)] = 0.049	Hydrogen site location: inferred from neighbouring sites
*wR*(*F*^2^) = 0.140	H atoms treated by a mixture of independent and constrained refinement
*S* = 1.02	*w* = 1/[σ^2^(*F*_o_^2^) + (0.0746*P*)^2^ + 0.2186*P*] where *P* = (*F*_o_^2^ + 2*F*_c_^2^)/3
5078 reflections	(Δ/σ)_max_ = 0.001
158 parameters	Δρ_max_ = 0.50 e Å^−^^3^
1 restraint	Δρ_min_ = −0.21 e Å^−^^3^

### Special details

**Table:**

Experimental. The data was collected with the Oxford Cyrosystem Cobra low-temperature attachment.
Geometry. All e.s.d.'s (except the e.s.d. in the dihedral angle between two l.s. planes) are estimated using the full covariance matrix. The cell e.s.d.'s are taken into account individually in the estimation of e.s.d.'s in distances, angles and torsion angles; correlations between e.s.d.'s in cell parameters are only used when they are defined by crystal symmetry. An approximate (isotropic) treatment of cell e.s.d.'s is used for estimating e.s.d.'s involving l.s. planes.
Refinement. Refinement of *F*^2^ against ALL reflections. The weighted *R*-factor *wR* and goodness of fit *S* are based on *F*^2^, conventional *R*-factors *R* are based on *F*, with *F* set to zero for negative *F*^2^. The threshold expression of *F*^2^ > σ(*F*^2^) is used only for calculating *R*-factors(gt) *etc*. and is not relevant to the choice of reflections for refinement. *R*-factors based on *F*^2^ are statistically about twice as large as those based on *F*, and *R*- factors based on ALL data will be even larger.

### Fractional atomic coordinates and isotropic or equivalent isotropic displacement parameters (Å^2^)

**Table:**

	*x*	*y*	*z*	*U*_iso_*/*U*_eq_	
O1	0.52173 (8)	0.67777 (7)	0.56764 (7)	0.02345 (16)	
H1O1	0.5435	0.6430	0.6395	0.035*	
O2	0.97943 (7)	1.10528 (6)	0.70095 (6)	0.01770 (14)	
O3	0.88217 (8)	1.12535 (7)	0.48165 (6)	0.02051 (14)	
N1	0.06716 (8)	0.30978 (7)	0.51061 (7)	0.01683 (15)	
N2	0.18939 (9)	0.28645 (8)	0.73537 (8)	0.02239 (17)	
H1N2	0.1354	0.2254	0.7348	0.027*	
H2N2	0.2550	0.3085	0.8082	0.027*	
C1	0.81342 (9)	0.89872 (8)	0.69825 (8)	0.01647 (16)	
H1A	0.8873	0.9180	0.7769	0.020*	
C2	0.72397 (10)	0.80096 (9)	0.69536 (8)	0.01740 (16)	
H2A	0.7387	0.7548	0.7714	0.021*	
C3	0.61195 (10)	0.77231 (9)	0.57782 (9)	0.01771 (16)	
C4	0.59084 (11)	0.84236 (10)	0.46429 (9)	0.02258 (19)	
H4A	0.5157	0.8243	0.3862	0.027*	
C5	0.68141 (10)	0.93876 (9)	0.46745 (9)	0.02087 (18)	
H5A	0.6673	0.9842	0.3910	0.025*	
C6	0.79383 (9)	0.96844 (8)	0.58454 (8)	0.01518 (15)	
C7	0.89111 (9)	1.07305 (8)	0.58888 (8)	0.01503 (15)	
C8	0.03894 (10)	0.36798 (9)	0.39292 (9)	0.01886 (17)	
H8A	−0.0348	0.3399	0.3183	0.023*	
C9	0.11667 (11)	0.46682 (9)	0.38189 (10)	0.02141 (18)	
H9A	0.0968	0.5068	0.3012	0.026*	
C10	0.22817 (11)	0.50629 (9)	0.49691 (10)	0.02254 (19)	
H10A	0.2836	0.5728	0.4919	0.027*	
C11	0.25595 (10)	0.44821 (9)	0.61585 (10)	0.02098 (18)	
H11A	0.3297	0.4750	0.6911	0.025*	
C12	0.17140 (10)	0.34698 (9)	0.62328 (9)	0.01766 (16)	
H1N1	0.0145 (17)	0.2453 (12)	0.5101 (19)	0.051 (5)*	

### Atomic displacement parameters (Å^2^)

**Table:**

	*U*^11^	*U*^22^	*U*^33^	*U*^12^	*U*^13^	*U*^23^
O1	0.0264 (4)	0.0269 (4)	0.0164 (3)	−0.0110 (3)	0.0069 (3)	0.0002 (3)
O2	0.0180 (3)	0.0203 (3)	0.0128 (3)	−0.0017 (2)	0.0032 (2)	−0.0020 (2)
O3	0.0219 (3)	0.0218 (3)	0.0143 (3)	−0.0048 (3)	0.0023 (2)	0.0040 (2)
N1	0.0170 (3)	0.0179 (3)	0.0144 (3)	−0.0029 (3)	0.0042 (3)	−0.0020 (3)
N2	0.0227 (4)	0.0254 (4)	0.0147 (3)	−0.0055 (3)	0.0015 (3)	−0.0015 (3)
C1	0.0167 (4)	0.0186 (4)	0.0127 (3)	−0.0001 (3)	0.0036 (3)	0.0001 (3)
C2	0.0189 (4)	0.0194 (4)	0.0136 (3)	−0.0011 (3)	0.0056 (3)	0.0016 (3)
C3	0.0192 (4)	0.0198 (4)	0.0151 (3)	−0.0037 (3)	0.0073 (3)	−0.0011 (3)
C4	0.0230 (4)	0.0288 (5)	0.0126 (3)	−0.0093 (4)	0.0024 (3)	0.0002 (3)
C5	0.0225 (4)	0.0245 (5)	0.0133 (3)	−0.0053 (3)	0.0036 (3)	0.0023 (3)
C6	0.0159 (3)	0.0164 (4)	0.0127 (3)	0.0000 (3)	0.0044 (3)	0.0002 (3)
C7	0.0154 (3)	0.0156 (4)	0.0129 (3)	0.0010 (3)	0.0037 (3)	0.0001 (3)
C8	0.0185 (4)	0.0218 (4)	0.0159 (4)	−0.0018 (3)	0.0057 (3)	−0.0008 (3)
C9	0.0226 (4)	0.0220 (4)	0.0206 (4)	−0.0025 (3)	0.0090 (3)	0.0011 (3)
C10	0.0210 (4)	0.0205 (4)	0.0276 (4)	−0.0052 (3)	0.0105 (4)	−0.0025 (3)
C11	0.0169 (4)	0.0213 (4)	0.0229 (4)	−0.0034 (3)	0.0049 (3)	−0.0046 (3)
C12	0.0162 (4)	0.0190 (4)	0.0163 (4)	−0.0005 (3)	0.0040 (3)	−0.0032 (3)

### Geometric parameters (Å, °)

**Table:**

O1—C3	1.3543 (11)	C3—C4	1.3957 (13)
O1—H1O1	0.8200	C4—C5	1.3861 (13)
O2—C7	1.2675 (10)	C4—H4A	0.93
O3—C7	1.2654 (10)	C5—C6	1.3990 (12)
N1—C12	1.3528 (11)	C5—H5A	0.93
N1—C8	1.3571 (12)	C6—C7	1.4956 (12)
N1—H1N1	0.881 (9)	C8—C9	1.3644 (13)
N2—C12	1.3338 (12)	C8—H8A	0.93
N2—H1N2	0.86	C9—C10	1.4099 (14)
N2—H2N2	0.86	C9—H9A	0.93
C1—C2	1.3908 (13)	C10—C11	1.3687 (14)
C1—C6	1.3980 (12)	C10—H10A	0.93
C1—H1A	0.93	C11—C12	1.4161 (13)
C2—C3	1.3975 (12)	C11—H11A	0.93
C2—H2A	0.93		
			
C3—O1—H1O1	109.5	C1—C6—C5	118.72 (8)
C12—N1—C8	122.35 (8)	C1—C6—C7	120.36 (8)
C12—N1—H1N1	121.1 (13)	C5—C6—C7	120.92 (8)
C8—N1—H1N1	116.5 (13)	O3—C7—O2	122.89 (8)
C12—N2—H1N2	120.0	O3—C7—C6	119.10 (7)
C12—N2—H2N2	120.0	O2—C7—C6	118.00 (7)
H1N2—N2—H2N2	120.0	N1—C8—C9	121.30 (9)
C2—C1—C6	120.90 (8)	N1—C8—H8A	119.3
C2—C1—H1A	119.5	C9—C8—H8A	119.3
C6—C1—H1A	119.5	C8—C9—C10	117.75 (9)
C1—C2—C3	119.83 (8)	C8—C9—H9A	121.1
C1—C2—H2A	120.1	C10—C9—H9A	121.1
C3—C2—H2A	120.1	C11—C10—C9	120.96 (9)
O1—C3—C4	117.49 (8)	C11—C10—H10A	119.5
O1—C3—C2	122.94 (8)	C9—C10—H10A	119.5
C4—C3—C2	119.57 (8)	C10—C11—C12	119.44 (9)
C5—C4—C3	120.30 (8)	C10—C11—H11A	120.3
C5—C4—H4A	119.9	C12—C11—H11A	120.3
C3—C4—H4A	119.9	N2—C12—N1	118.41 (8)
C4—C5—C6	120.67 (8)	N2—C12—C11	123.43 (8)
C4—C5—H5A	119.7	N1—C12—C11	118.17 (8)
C6—C5—H5A	119.7		
			
C6—C1—C2—C3	−0.63 (14)	C5—C6—C7—O3	−8.78 (13)
C1—C2—C3—O1	179.36 (9)	C1—C6—C7—O2	−8.40 (12)
C1—C2—C3—C4	−0.07 (14)	C5—C6—C7—O2	171.26 (9)
O1—C3—C4—C5	−178.61 (9)	C12—N1—C8—C9	1.07 (14)
C2—C3—C4—C5	0.84 (15)	N1—C8—C9—C10	0.35 (14)
C3—C4—C5—C6	−0.92 (16)	C8—C9—C10—C11	−0.87 (15)
C2—C1—C6—C5	0.55 (13)	C9—C10—C11—C12	0.02 (15)
C2—C1—C6—C7	−179.78 (8)	C8—N1—C12—N2	178.22 (9)
C4—C5—C6—C1	0.22 (14)	C8—N1—C12—C11	−1.90 (14)
C4—C5—C6—C7	−179.44 (9)	C10—C11—C12—N2	−178.79 (9)
C1—C6—C7—O3	171.56 (8)	C10—C11—C12—N1	1.33 (14)

### Hydrogen-bond geometry (Å, °)

**Table:**

*D*—H···*A*	*D*—H	H···*A*	*D*···*A*	*D*—H···*A*
O1—H1O1···O2^i^	0.82	1.86	2.6257 (9)	154
N2—H1N2···O2^ii^	0.86	1.98	2.8224 (11)	167
N2—H2N2···O3^iii^	0.86	1.99	2.8396 (11)	171
N1—H1N1···O3^ii^	0.88 (1)	1.81 (1)	2.6861 (10)	169 (2)
C10—H10A···O1	0.93	2.51	3.3482 (14)	149
C11—H11A···O2^i^	0.93	2.34	3.1899 (12)	152

Symmetry codes: (i) −*x*+3/2, *y*−1/2, −*z*+3/2; (ii) *x*−1, *y*−1, *z*; (iii) *x*−1/2, −*y*+3/2, *z*+1/2.
